# Mitochondrial dysfunction induced by frataxin deficiency is associated with cellular senescence and abnormal calcium metabolism

**DOI:** 10.3389/fncel.2014.00124

**Published:** 2014-05-13

**Authors:** Arantxa Bolinches-Amorós, Belén Mollá, David Pla-Martín, Francesc Palau, Pilar González-Cabo

**Affiliations:** ^1^Program in Rare and Genetic Diseases, Centro de Investigación Príncipe FelipeValencia, Spain; ^2^IBV/CSIC Associated Unit, Centro de Investigación Príncipe FelipeValencia, Spain; ^3^CIBER de Enfermedades RarasValencia, Spain; ^4^Facultad de Medicina de Ciudad Real, Universidad de Castilla-La ManchaCiudad Real, Spain

**Keywords:** Friedreich ataxia, frataxin, mitochondrial dysfunction, cellular senescence, autophagy, calcium metabolism, ER-stress

## Abstract

Friedreich ataxia is considered a neurodegenerative disorder involving both the peripheral and central nervous systems. Dorsal root ganglia (DRG) are the major target tissue structures. This neuropathy is caused by mutations in the *FXN* gene that encodes frataxin. Here, we investigated the mitochondrial and cell consequences of frataxin depletion in a cellular model based on frataxin silencing in SH-SY5Y human neuroblastoma cells, a cell line that has been used widely as *in vitro* models for studies on neurological diseases. We showed that the reduction of frataxin induced mitochondrial dysfunction due to a bioenergetic deficit and abnormal Ca^2+^ homeostasis in the mitochondria that were associated with oxidative and endoplasmic reticulum stresses. The depletion of frataxin did not cause cell death but increased autophagy, which may have a cytoprotective effect against cellular insults such as oxidative stress. Frataxin silencing provoked slow cell growth associated with cellular senescence, as demonstrated by increased SA-βgal activity and cell cycle arrest at the G1 phase. We postulate that cellular senescence might be related to a hypoplastic defect in the DRG during neurodevelopment, as suggested by necropsy studies.

## INTRODUCTION

Friedreich ataxia (FRDA) is the most common autosomal recessive ataxia, with a prevalence of two to four individuals per 100,000 in Caucasian populations (Lopez-Arlandis et al., 1995; Vankan, 2013). Both the peripheral and central nervous systems are involved. Neuropathology and clinical pictures are the consequences of sensory axonal neuropathy characterized by degeneration of the large sensory neurons at the dorsal root ganglia (DRG), spinocerebellar tracts, and corticospinal tracts, and degeneration of cerebellar deep nuclei, namely the dentate nucleus. Hypertrophic cardiomyopathy and increased incidence in diabetes and skeletal deformities are also characteristic of the disease (Harding, 1981; Koeppen and Mazurkiewicz, 2013; Weidemann et al., 2013). Mutations in the *FXN* gene cause FRDA. *FXN* maps to chromosome 9q13 and encodes frataxin, a small protein of 210 amino acids (Campuzano et al., 1996) associated with the mitochondrial inner membrane (Babcock et al., 1997; Campuzano et al., 1997; Priller et al., 1997; Koutnikova et al., 1998). Pathophysiology of the disease is due to the reduced amount of frataxin in targeted neural and non-neural cells and tissues (Deutsch et al., 2010).

A number of physiological functions for frataxin in mitochondria have been proposed; the most accepted role is in the biogenesis of iron-sulfur clusters (ISC; Gerber et al., 2003; Ramazzotti et al., 2004), but other functions such as the metabolism of mitochondrial iron and the response to oxidative stress (Babcock et al., 1997; Foury and Cazzalini, 1997; Wilson and Roof, 1997), an iron-storage protein maintaining iron in a non-toxic and bioavailable form (Adamec et al., 2000; Park et al., 2003), maturation of heme-containing proteins (Lesuisse et al., 2003; Yoon and Cowan, 2004), and mitochondrial energy conversion and oxidative phosphorylation (Ristow et al., 2000; Gonzalez-Cabo et al., 2005) have been proposed as well. The lack of frataxin causes mitochondrial dysfunction (Vazquez-Manrique et al., 2006; Llorens et al., 2007; Gonzalez-Cabo and Palau, 2013), which has a direct effect on the pathophysiology of the disease. Proper mitochondrial function is essential for the neuronal survival by different physiological functions such as energy production, maintenance of membrane potential, regulation of cellular Ca^2+^ homeostasis, protein folding by chaperones, dendritic and axonal transport, and release and reutilization of synaptic neurotransmitters. Due to the variety of functions that the mitochondria perform, it is not surprising that mitochondrial dysfunction has severe consequences at the cellular level, which are intimately related to aging and neurodegenerative diseases (Kwong et al., 2006; Tatsuta and Langer, 2008).

Here, we present the cellular and mitochondrial consequences of frataxin deficiency in a cellular model based on gene silencing in the human neuroblastoma cell line SH-SY5Y. Neuroblastoma is a developmental tumor originated from the neural crest, like DRG neurons. This shared origin makes neuroblastoma cell lines a good cellular model to study disorders related to DRG and other neural crest-derived cells. We have observed cellular senescence and mitochondrial dysfunction associated with low energy production and abnormal Ca^2+^ homeostasis, oxidative and endoplasmic reticulum (ER) stresses, and an increase of autophagy. The senescence phenotype could be involved in the neurodegeneration and abnormal development in the FRDA pathogenesis. The present study, therefore, implicates calcium homeostasis, ER stress, and cellular senescence as potential contributing factors in FRDA. We propose these phenomena as new drug and neuroprotection targets.

## MATERIALS AND METHODS

### CELL CULTURE AND PRODUCTION OF STABLE SH-SY5Y CELL LINES

The human SH-SY5Y neuroblastoma cell line was grown in DMEM-F12 (Gibco, Invitrogen) supplemented with 10% fetal bovine serum containing 2 mM L-glutamine and antibiotics, and maintained at 37°C in an atmosphere of 5% CO_2_ in air.

For the generation of stable cell lines with gene silencing of *FXN*, SH-SY5Y cells were transfected with pLKO.1 vector (MISSION®shRNA plasmid DNA, Sigma-Aldrich) containing a hairpin sequence of *FXN* (TRCN0000006138). Control cells were transfected with non-target control vector. Transfections were performed using SuperFect Transfection (Qiagen) according to the manufacturer’s instructions. The stably transfected cells were selected and maintained in medium with 2 μg/ml puromycin.

### WESTERN BLOTTING

Cells were harvested and centrifuged (100 × *g*, 5 min), and the cell pellets were re-suspended in lysis buffer (50 mM TrisHCl, pH 7.5; 10 mM NaCl; 50 mM EDTA, pH 8; 15% glycerol) containing protease inhibitors (Roche). After 30 min of incubation on ice, cell lysates were subjected to three cycles of freeze-thawing in liquid nitrogen. Next, the lysates were centrifuged (15,000 x *g*, 4°C, 10 min) and the supernatant was collected to use for detection of frataxin expression. Protein extracts of the supernatant were resolved by SDS-PAGE and transferred to PVDF membrane. Membranes were stained with specific antibodies: frataxin (Immunological Sciences), LC3 (Sigma), COX1 (Mitosciences), and COX2 (Molecular Probes), catalase (Sigma), SOD 2 (Abnova), SOD 1 (Abnova), cytochrome *c* (BD Biosciences), caspase-3 (Cell Signaling), BIP (cell Signaling), actin (Sigma), and OPA1 (BD Biosciences) antibodies. Equal loading was assessed using an antibody against actin (Sigma). After incubation with the appropriate secondary antibodies, protein bands were detected using a Fujifilm Las-3000 after incubation with the ECL Plus Western Blotting Detection System (GE Healthcare). Density of the bands was quantified by Multi Gauge V2.1 software.

### CELLULAR GROWTH CURVE CONTROL

Cells in log phase were trypsinized and seeded in six-well plates at a density of 25,000 cells/well. The cells were trypsinized and counted every 24 h for 13 successive days in order to complete the growth curve in vitro.

### EVALUATION OF CELL CYCLE

Samples containing 1 × 10^6^ cells were harvested by centrifugation at 250 × *g* for 10 min at 4°C and fixed with 70% cold ethanol for at least 2 h at -20°C. Cells were harvested by centrifugation at 2700 × *g* for 10 min at 4°C and resuspended in 1 ml of PBS with 0.05 mg/ml propidium iodide (PI) and 0.25 mg/ml RNase. The cells were incubated for 30 min at 37°C. Cell cycle profiles (30,000 cells) were analyzed by a FACS Canto flow cytometer (BD) with an FL2 detector. FACS Diva software (BD) was used for analysis of cell cycle phase.

### SENESCENCE-ASSOCIATED BETA-GALACTOSIDASE (SA-βGAL) ASSAY

Cells were seeded in a six-well plate at 5000 cells/cm^2^. At 24 h, the cells were incubated for 2 h with 100 nM bafilomycin A1 to inhibit lysosomal beta-galactosidase. After the wells were washed with 1× PBS, the cells were fixed with 0.2% glutaraldehyde for 10 min at room temperature, washed three times with 1x PBS, and incubated for 6 h at 37°C in the dark with staining solution (5 mM C_6_N_6_FeK_4_, 5 mM C_6_N_6_FeK_3_, 2 mM MgCl_2_, 1 mg/mL X-gal, pH 6.0). Blue cells were counted under a microscope.

### MITOCHONDRIAL RESPIRATORY CHAIN ACTIVITIES

Mitochondria were prepared from SH-SY5Y cells using the Mitochondria Isolation Kit (Sigma) following the manufacturer’s instructions. The final pellet represented a crude mitochondrial fraction. Freshly obtained mitochondria were assayed for complex I, complex II, complex III, complex IV, complex I+II, and complex II+III activities. All assays were measured as previously described (Gonzalez-Cabo et al., 2010).

### ATP ASSAYS

Cells were trypsinized and resuspended in 0.5 ml PBS (1 × 10^6^ cells/ml). ATP levels were determined using the Adenosine 5′-triphosphate (ATP) Bioluminescent Assay Kit (Sigma) following the manufacturer’s instructions. Each sample was measured in triplicate using Wallac Victor^2TM^ 1420 Multilabel Counter (Perkin Elmer).

### OXYGEN CONSUMPTION ASSAY

Oxygen consumption was assessed using a Clark-type oxygen electrode (Rank Brothers) at 37°C with a magnetic stirrer. Cells were suspended in 1 ml of Hank’s Balanced Salt Solution (Invitrogen). Oxygen consumption is given in nmol O_2_ min^-1^ 10^6^ cells^-1^.

### MITOCHONDRIAL TRANSMEMBRANE GRADIENT (ΔΨ_M_)

Suspended cells for all the clones (8 × 10^5^ cells/ml) were incubated at 37°C for 30 min with JC-1 dye (5 μg/ml; Molecular Probes, Invitrogen). Appropriate uncoupling controls were set up by treating the cells with 500 μM of the uncoupler carbonyl cyanide *m*-chlorophenylhydrazine (CCCP) at 37°C for 5 min. ΔΨ_M_ was determined by flow cytometry using the BD FACS Canto. The emission of JC-1 monomers and aggregates was detected through 530 ± 15 and 585 ± 20 nm filters, respectively. The data were analyzed using BD FACS Diva software. This experiment was repeated at least seven times.

### OXIDATIVE STRESS ASSAYS

To analyze protein carbonylation, we used the Oxyblot^TM^ Protein Oxidation Detection Kit (Millipore). Briefly, a cell lysate containing 15 μg of protein, prepared as described above, was incubated in 12% SDS supplemented with 2,4-dinitrophenylhydrazine (DNPH) for 10 min at room temperature. Samples were resolved by western blotting using an anti-DNP antibody.

The detection of superoxide in live cells was performed using MitoSOX^TM^ Red superoxide indicator (Invitrogen). Cells were seeded directly on glass coverslips in six-well plates at a density of 20,000 cells/well. The positive control, pLKO.1-NT cells, were treated with 50 μM H_2_O_2_ overnight. The rest of the cell lines did not receive this treatment. The next day, the cells were incubated with PBS containing 3.75 μM MitoSOX^TM^ for 10 min at 37°C in the dark. Nuclei were stained with DAPI (Sigma). The coverslips were then fixed on microscope slides and digitized with a Hamamatsu camera (Tokyo, Japan) connected to a Leica DMR microscope (Nussloch, Germany).To quantify superoxide production, we collected fluorescence images of more than 200 cells for each clone. We then measured the pixels produced by fluorescence in the cells and determined the fluorescence level relative to cell area, using ImageJ.

### MEASUREMENT OF APOPTOSIS

Wild-type SH-SY5Y cells were irradiated at room temperature by a crosslinker with doses of 8 J/cm^2^ during 30 s. For Western blotting, cells were recovered within16 h after exposure to the radiation. The rest of the cell lines did not receive this treatment.

Cytochrome *c* release from the mitochondria and active caspase-3 were measured in SH-SY5Y cell lines for 72 h and 6 days. Cell fractions were obtained (cytosolic and mitochondrial) and then protein extracts of the cell fractions were tested by Western blotting using cytochrome *c* (BD Biosciences) and caspase-3 (Cell Signaling) antibodies. Equal loading was assessed using antibodies against actin (Sigma) and OPA1 (BD Biosciences).

### AUTOPHAGY PROCESS MEASUREMENTS

SH-SY5Y cells were transfected with pEGFP-LC3 using the lipofectamine reagent (Invitrogen) according to the manufacturer’s instructions. After 24 h of transfection, the cells were analyzed by fluorescence microscopy.

Autophagy was induced in SH-SY5Y cells cultured in DMEM-F12 without fetal bovine serum overnight. To inhibit autophagy, the cells were treated with different pharmacological or molecular inhibitors: 0.1 μM of bafilomycin A1 (Sigma) for 2 h (inhibitor of the late phase of autophagy), 100 nM insulin (Sigma) for 90 min, or 1 μM phorbol 12-myristate 13-acetate (PMA, Sigma) for 2 h. After the treatments, the cells were harvested and lysed to test variations in LC3-II levels by Western blotting.

### ENDOPLASMIC RETICULUM STRESS

Susceptibility to ER stress was assessed as described by Vernia et al. (2009). Briefly, SH-SY5Y cells were treated with 1 μM thapsigargin (Alomone Labs) and were maintained in culture during 18 h before analysis by Western blotting.

To analyze apoptosis, samples containing 1 × 10^6^ treated cells were harvested by centrifugation at 250 × *g* for 10 min at 4°C and fixed with 70% cold ethanol for at least 2 h at -20°C. Cells were harvested by centrifugation at 2700 × *g* for 10 min at 4°C and resuspended in 1 ml PBS with 0.05 mg/ml PI and 0.25 mg/ml RNase. The cells were incubated for 30 min at 37°C. Cell cycle profiles (30,000 cells) were analyzed by a FACS Canto flow cytometer (BD) with an FL2 detector. FACS Diva software (BD) was used for analysis of cell cycle phase.

### MEASUREMENTS OF [Ca^2+^]_CYT_ AND MITOCHONDRIAL Ca^2+^ UPTAKE

Cytosolic calcium imaging with Fura-2 was performed as described by Pla-Martin et al. (2013). Changes in fluorescence ratio were recorded after the addition of 25 μM veratridine (Sigma) in 2 mM CaCl_2_–HCSS medium or 100 μM 2,5-di-(ter-butyl)-1,4-benzohydroquinone (tBuBHQ, Alomone Labs) in Ca^+2^-free HCSS medium. To activate store-operated calcium entry (SOCE) induced by ER calcium release with tBuBHQ, 2 mM CaCl_2_ was added. Images were analyzed using Leica MM Fluor software.

To study mitochondrial calcium uptake and swelling, we adapted the protocol described by Traba et al. (2012). The experiments were carried out in a Wallac Victor2^TM^ 1420 Multilabel Counter (PerkinElmer) at 30°C in the presence of 5 mM succinate, 1 μM rotenone, and 100 μM ADP. Mitochondrial calcium uptake was assessed by monitoring the decrease in Calcium Green-5N fluorescence at 535 nm every 7 min after adding pulses of 4 mM CaCl_2_.

### MEASURING MITOCHONDRIAL LENGTH AND NUMBER

Cells were probed with anti-cytochrome *c* antibody to determine mitochondrial length and number. To obtain quantitative data, a number of cells (*n* = 14–31) were typically imaged. Morphological measurements were made using ImageJ by automated object identification with user-defined thresholds for pixel intensity and size. Objects were skeletonized and measurements of length were obtained.

### MICROSCOPY STUDIES

Cells were seeded directly on glass coverslips. Twenty-four hours later, they were fixed with 2 and 4% paraformaldehyde solution successively and then permeabilized with a solution of 0.5% PBS-Triton X-100. Cells were probed with anti-cytochrome c antibody (Zymed) in blocking solution (PBS/3% FBS) and then with fluorescent Alexa Fluor 488 (Molecular probes). Similarly, cells were stained with DAPI (Sigma) to visualize nuclei. Appropriate negative controls were set up by incubating fixed cells with secondary antibodies only. Coverslips were then fixed on microscope slides and digitized with a Hamamatsu camera (Tokyo, Japan) connected to a Leica DMR microscope (Nussloch, Germany). All images were captured under constant exposure time, gain, and offset. We defined four different mitochondrial architectures in live cells: tubular (long and higher interconnectivity), mixed (mixture of tubular and vesicular), vesicular (short tubular segments), and fragmented (small and round). Mitochondrial morphology evaluation was performed by a researcher blinded to genotype. At least 100 cells were counted and the average was calculated.

### ELECTRON MICROSCOPY

Cells were seeded at 2000 cells/cm^2^ in Lab-Tek chamber slides of two wells (Nalge Nunc International, Naperville, IL, USA) and were fixed in 2.5 % glutaraldehyde for 1 h at 37°C. Subsequently, a standard protocol (Marcilla-Etxenike et al., 2012) was applied in these cells to obtain ultrathin sections. Photomicrographs were obtained under a transmission electron microscope FEI Tecnai G2 Spirit (FEI Europe) using a digital camera Morada (Olympus Soft Image Solutions GmbH). At least 20 cells of each clone were analyzed.

## RESULTS

### SILENCING OF FXN EXPRESSION INDUCES CELLULAR SENESCENCE BUT NOT APOPTOSIS

**FIGURE 1:**
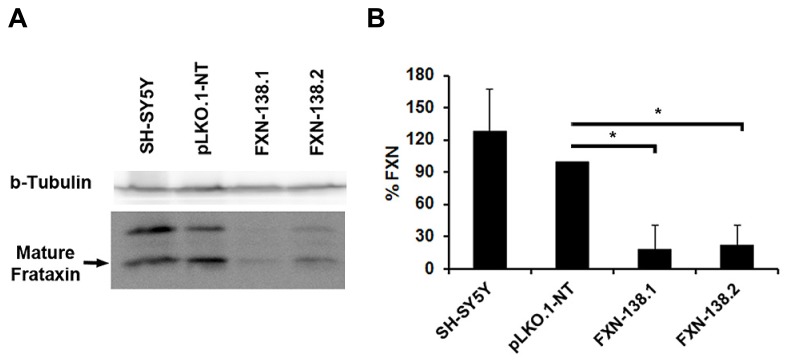
**Depletion of frataxin by siRNA.** SH-SY5Y cells were transfected with the vector pLKO-FXN. Two SH-SY5Y stable clones exhibiting the most efficient silencing were selected, FXN-138.1 and FXN-138.2. **(A)** Cell lysates were analyzed by SDS-PAGE and immunoblotting for frataxin (bottom panel; upper and lower bands correspond to intermediate and mature forms of frataxin, respectively). Beta-tubulin was used as a loading control (top panel). **(B)** Bands corresponding to mature frataxin were quantified and final values are expressed as a percentage of the pLKO.1-NT value. Student’s t-test, FXN-138.1 p = 0.025, FXN-138.2 p=0.019 versus pLKO.1-NT. *p ≤ 0.05.

**FIGURE 2:**
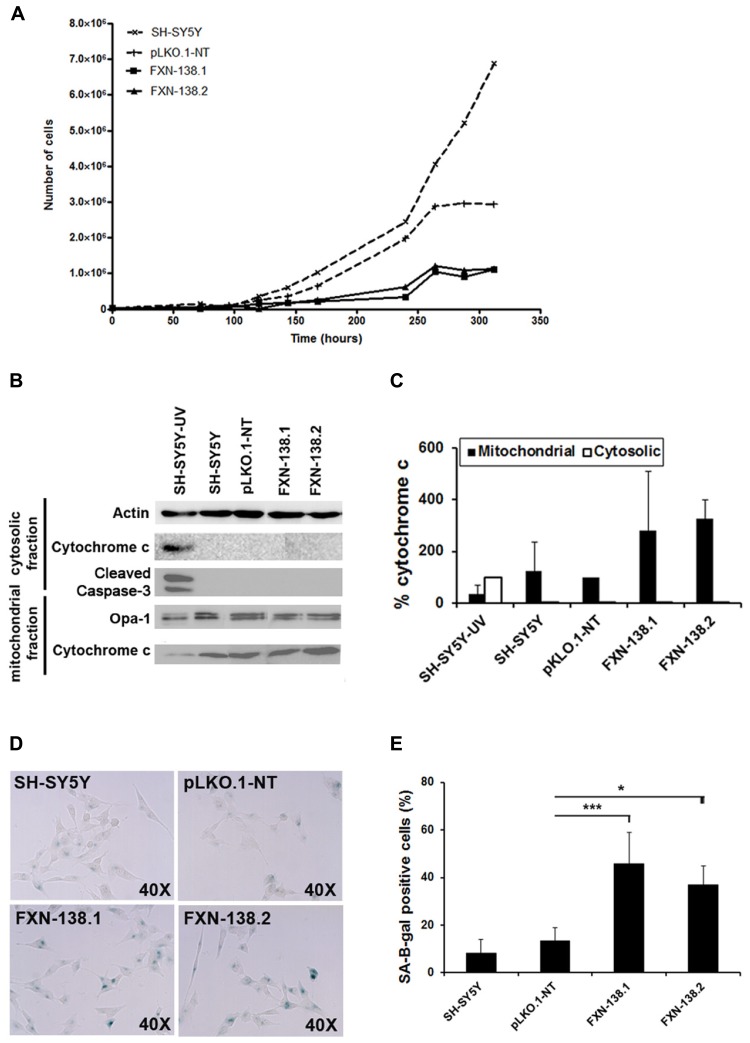
**Increased cellular senescence response in *FXN*-deficient clones. (A)** Growth curves of SH-SY5Y (wild-type), pLKO.1-NT, FXN-138.1, and FXN-138.2 cells. The cells were trypsinized and counted every 24 h for 13 successive days. Data are expressed as the mean of three experiments. **(B)** Western blot analysis of extracts from cells after 6 days of culture from untreated wild-type (WT) SH-SY5Y, pLKO.1-NT, FXN-138.1, and FXN-138.2 cells and WT SH-SY5Y cells exposed to radiation (apoptotic control), using cleaved caspase-3 antibody and cytochrome *c* antibody. Only the apoptotic control showed caspase-3 activation. All clones retained cytochrome *c* in the mitochondria, which is consistent with a non-apoptotic state, while the apoptotic control showed cytochrome *c* release into the cytosol. **(C)** A quantitative Western blot assay was developed to measure total cytochrome *c* in the mitochondria and cytosol. Data from cytosolic cytochrome c are expressed as percentage relative to apoptotic control, whereas data from mitochondrial cytochrome c are expressed as percentage relative to pLKO.1-NT control. **(D)** The clones indicated in the pictures were subjected to in situ SA-β-gal staining (blue) and examined by bright field microscopy. (**E**) The figure represents the expression level of the lysosomal enzyme β-galactosidase in all lines. The columns and bar show the mean and standard deviation of no fewer than 1300 cells from at least three experiments. Student’s *t*-test, FXN-138.1 *p* = 0.001, FXN-138.2 *p* = 0.02 versus pLKO.1-NT. *p ≤ 0.05; ***p ≤ 0.001.

To investigate the consequences of frataxin depletion in cells and mitochondria, we generated *FXN*-silenced clones of the human neuroblastoma SH-SY5Y cell line using five different shRNA targeted to five different sites on the *FXN* gene. Clones FXN-138.1 and FXN-138.2 (82 and 78% frataxin reduction, respectively) were used for further experiments (**Figure 1**). We observed that the proliferation of both silenced clones was limited compared with both the WT neuroblastoma cells and the non-targeted shRNA clone (pLKO.1-NT) used as a negative control, in a time-dependent manner (**Figure 2A**). Limited proliferation was confirmed by flow cytometry that showed significant reduced progression to the G2/M and S phase and growth arrest at G1 in knockdown cells (**Table 1**). We did not detect a loss of cell viability in silenced clones, suggesting that the depletion of frataxin did not induce cell death.

**Table 1:** Distribution of cell cycle phases (%) of wild-type (WT) SH-SY5Y, pLKO.1-NT, FXN-138.1, and FXN-138.2 cells.

	WT SH-SY5Y	pLKO.1-NT	FXN-138.1	FXN-138.2
SubG0	1.63 ± 0.38	2.90 ± 0.44	2.20 ± 0.50	6.07 ± 4.05
G1	64.60 ± 7.20	70.93 ± 3.65	82.73 ± 1.69^*^	76.17 ± 7.33
S	22.70 ± 6.33	16.13 ± 3.88	8.5 ± 1.21^#^	10.77 ± 5.35
G2/M	10.27 ± 0.95	9.17 ± 0.84	5.87 ± 0.45^&^	6.43 ± 1.43^¥^

Student’s *t*-test, ^*^p = 0.007; ^#^*p* = 0.031; ^&^*p* = 0.004; ^¥^*p* = 0.046.

Furthermore, neither FXN-138.1 nor FXN-138.2 expressed cleaved caspase-3 (**Figure 2B**), nor did they show increased levels of cytosolic cytochrome *c* (**Figures 2B,C**) in basal conditions after 72 h and 6 days of culture, which suggested that the lack of frataxin in the SH-SY5Y cells did not induce apoptosis. Then, we investigated cellular senescence in the knockdown cells by measuring the senescence-associated β-galactosidase (SA-β-gal) activity (**Figure 2D**). We observed a significant increase of SA-β-gal positive cells (Student’s *t*-test, FXN-138.1 *p* = 0.001, FXN-138.2 *p* = 0.02) which confirmed the senescent phenotype of cells with reduced amounts of frataxin (**Figure 2E**).

### DEPLETION OF FRATAXIN AFFECTS MITOCHONDRIAL BIOENERGETICS AND INDUCES OXIDATIVE STRESS

**FIGURE 3:**
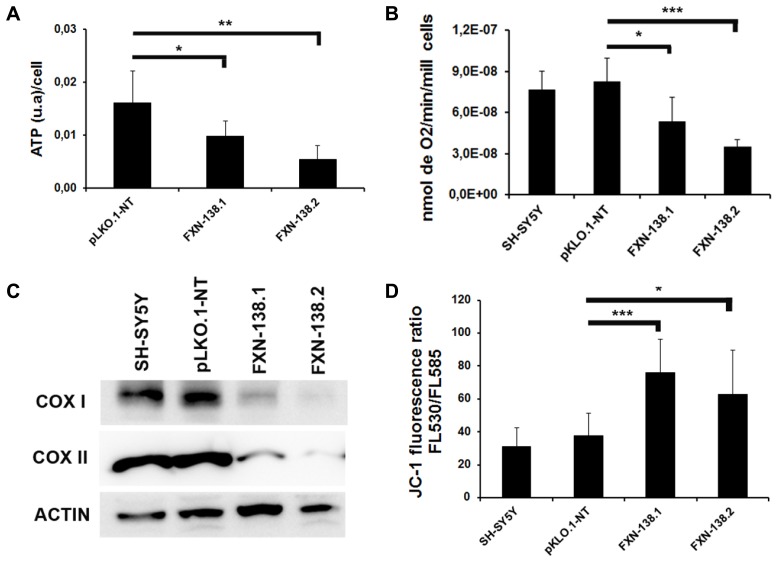
**Alterations in mitochondrial bioenergetics in frataxin-deficient cells. (A)** ATP synthesis was measured in all the clones and was reduced in frataxin-deficient cells. Error bars indicate the standard deviation (SD) of at least three independent assays. Student’s *t*-test, FXN-138.1 *p* = 0.034, FXN-138.2 *p* = 0.003 versus pLKO.1-NT. **(B)** Oxygen consumption was assessed using a Clark-type oxygen electrode. Oxygen consumption was lower in clones deficient in frataxin. Student’s *t*-test, FXN-138.1 *p* = 0.035, FXN-138.2 *p* = 0.00004 versus pLKO.1-NT. **(C)** Western blot analysis of Complex IV of mitochondrial extracts from all the cells confirmed less COX1 and COX2 protein in frataxin-deficient clones, which correlated with results of Complex IV activity. Results are representative of at least three experiments with similar results. **(D)** Quantification of mitochondrial membrane potential in SH-SY5Y clones analyzed by FACS. Shown in this figure is the ratio of green/red fluorescence for the different clones. We observed depolarization of membrane potential in *FXN*-deficient clones. Student’s *t*-test, FXN-138.1 *p* = 0.001, FXN-138.2 *p* = 0.050 versus pLKO.1-NT. The columns and bar show the mean and SD of at least three experiments. *p ≤ 0.05; **p ≤ 0.01; ***p ≤ 0.001.

**FIGURE 4:**
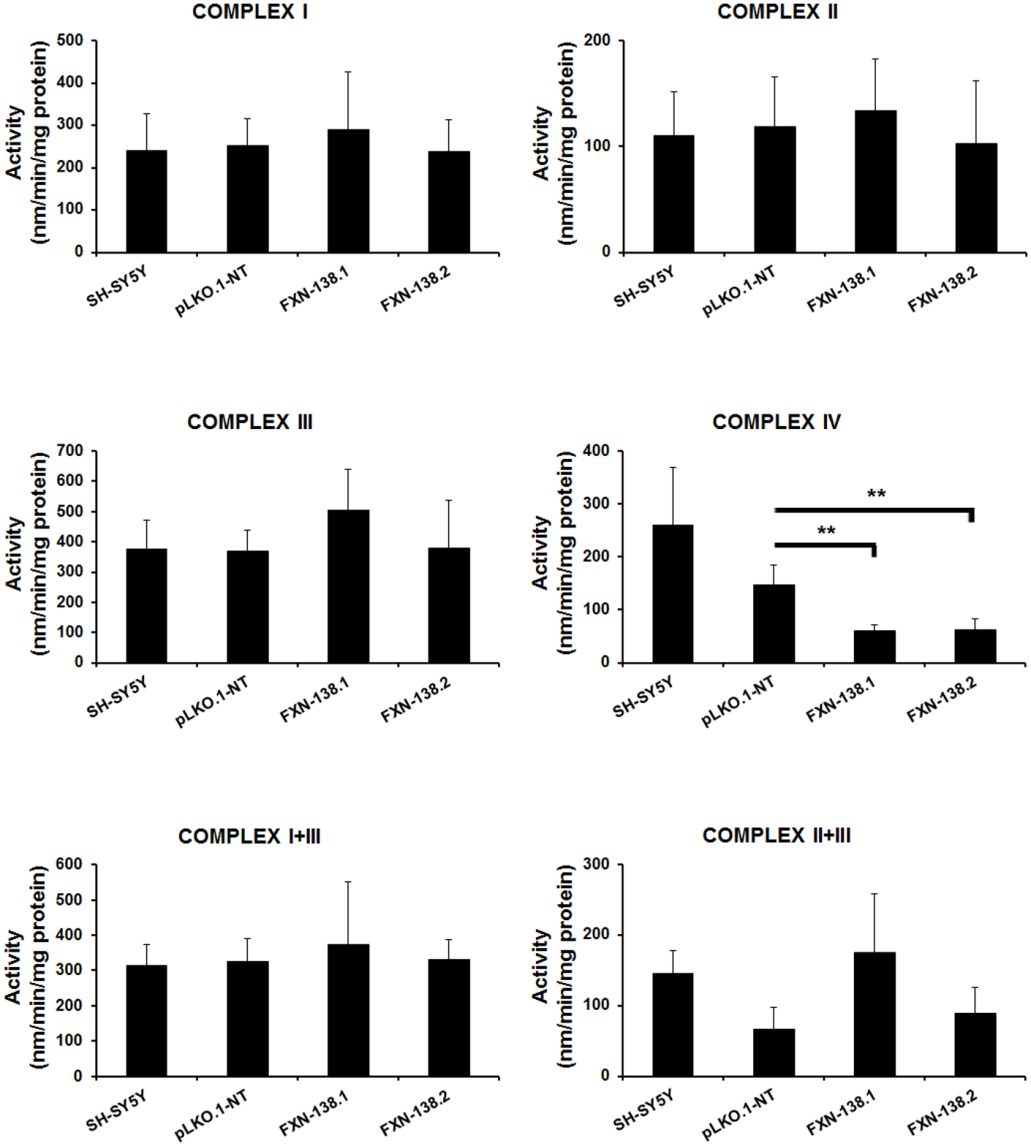
**Enzymatic activity analysis of the ETC in SH-SY5Y clones.** Wild-type SH-SY5Y, pLKO.1-NT, FXN-138.1, and FXN-138.2 cells were grown for 1 month. Mitochondria were isolated and used to determine the enzymatic activity of Complex I, II, III, IV, I+II, and I+III of the respiratory chain. Error bars indicate the standard deviation of at least three independent measurements (Student’s *t*-test, FXN-138.1 *p* = 0.0063, FXN-138.2 *p* = 0.002 versus pLKO.1-NT. **p ≤ 0.01).

We investigated the bioenergetic metabolism of *FXN*-silenced clones in cell culture for 1 month by measuring oxidative phosphorylation (OXPHOS), electron transport chain (ETC) complexes, and mitochondrial membrane potential. We observed a significant reduction of ATP production (**Figure 3A**) and a decrease of oxygen consumption (**Figure 3B**) in *FXN*-depleted clones, which suggested a defect in energy metabolism. Then, we measured the enzymatic activities of ETC complexes. The depletion of frataxin was associated with the loss of complex IV activity (**Figure 4**). The loss of enzymatic activity was correlated with a reduction in the amount of two complex IV subunits, cytochrome *c* oxidase subunits 1 and 2 (COX1 and COX2; **Figure 3C**). Mitochondrial inner membrane potential (ΔΨm) was also altered, as the JC-1 label showed a significant increase of the JC-1 green/red ratio, indicating depolarization of the mitochondrial membrane in both *FXN*-silenced clones compared with pLKO.1-NT control cells (**Figure 3D**). Taken as a whole, these findings indicated that silencing of the *FXN* gene in SH-SY5Y cells affected energy metabolism and suggested that the abnormal mitochondrial function was the consequence of the partial loss of frataxin.

**FIGURE 5:**
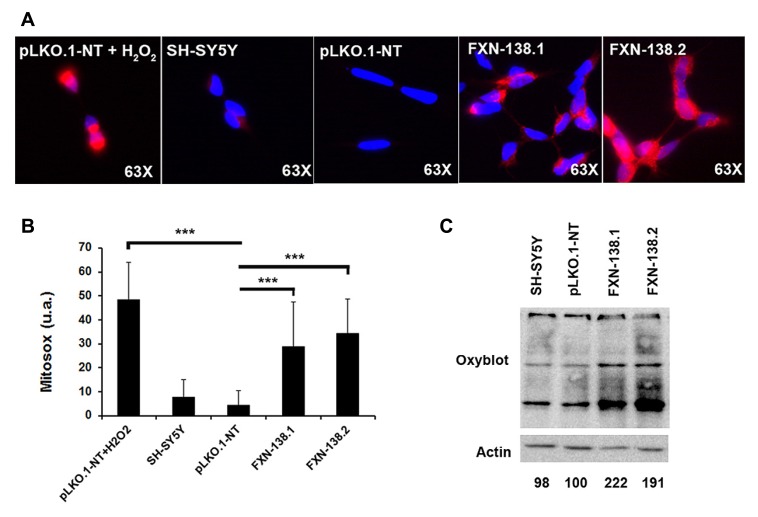
**Detection of protein oxidation and superoxide abundance. (A)** Detection of superoxide in live cells of SH-SY5Y clones using MitoSox^TM^ Red superoxide indicator. Representative images of MitoSOX stained cells fromSH-SY5Y, pLKO.1-NT, FXN-138.1, and FXN-138.2. pLKO.1-NT was treated with or without H_2_O_2_. **(B)** Quantitative analysis of MitoSOX red fluorescence intensity. Fluorescence level is presented relative to cell area in every clone. Student’s *t*-test, FXN-138.1 *p* = 1.8 × 10^-7^, FXN-138.2 *p* = 6 × 10^-14^ versus pLKO.1-NT. **(C)** Oxyblot^TM^ assay on wild-type SH-SY5Y, pLKO.1-NT, FXN-138.1, and FXN-138.2 cells. Carbonylated proteins were quantified for each lane using Fujifilm’s Multi-Gauge Software. To allow for loading variation, values were normalized to the actin control. Final values are expressed as a percentage of pLKO.1-NT value and shown below each lane. It can be seen that FXN-138.1 and FXN-138.2 cells showed a marked increase in carbonylated proteins, demonstrating direct evidence of cellular oxidative stress. ***p ≤ 0.001.

**FIGURE 6:**
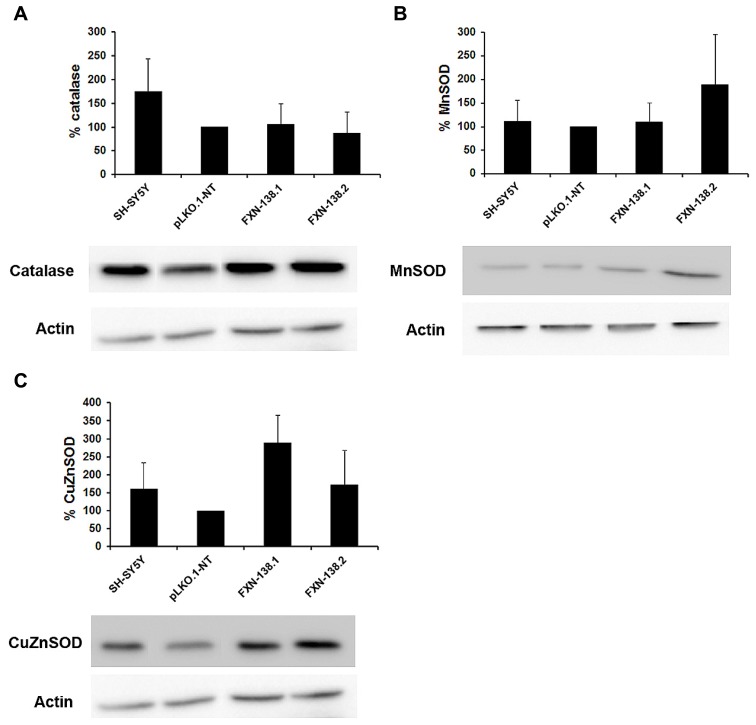
**Analysis of antioxidant enzymes.** Western blots analyses of antioxidant enzymes were carried out. Once normalized the intensities, the protein levels of antioxidant enzymes are expressed as percentages versus control pLKO.1-NT: **(A)** catalase, **(B)** MnSOD, and **(C)** CuZnSOD. The columns and bar represent the mean and standard deviation of at least three experiments.

Mitochondria are the principal source of reactive oxygen species (ROS) within the cell, including the superoxide radical anion ($O_{2}^{•-}$) and hydrogen peroxide (H_2_O_2_). Mitochondrial OXPHOS dysfunction may increase ROS production, with a damaging effect on these organelles. Frataxin knockdown cells showed a reduction of complex IV activity and reduced levels of COX1 and COX2 subunits. To determine whether frataxin down regulation induced oxidative stress, we investigated the production of $O_{2}^{•-}$ and the effects of increased free radical levels on proteins. The quantitation of $O_{2}^{•-}$ by the MitoSOX^TM^ probe showed a significant increase in frataxin-depleted clones against pLKO.1-NT control cells (**Figures 5A,B**; Student’s *t*-test, FXN-138.1 *p* = 1.8 × 10^-7^, FXN-138.2 *p* = 6 × 10^-14^). As positive control we used pLKO.1-NT cells treated with H_2_O_2_. Proteins showed the tendency for carbonylation in silenced clones (**Figure 5C**), suggesting again that an effect of ROS was associated with frataxin depletion. Such an increase in oxidative stress, however, did not induce any specific response of antioxidant defenses, as protein expression of superoxide dismutases, MnSOD and CuZnSOD, and catalase was not significantly modified (**Figure 6**).

### DEFICIT OF FRATAXIN INDUCES MITOCHONDRIAL FUSION

**FIGURE 7:**
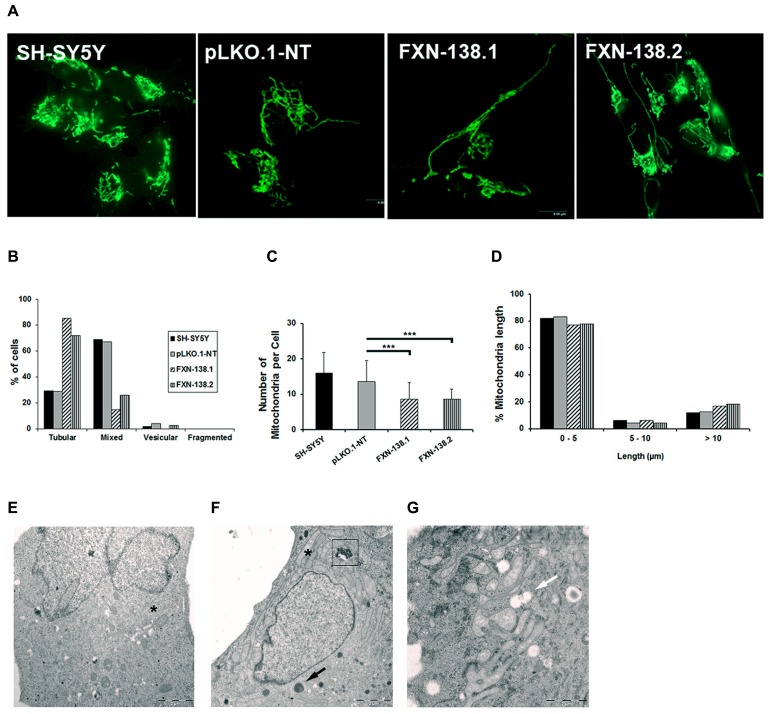
**Quantification of mitochondrial dynamic process in SH-SY5Y clones. (A)** Representative mitochondrial network shows increased fusion morphology in frataxin-deficient cells. **(B)** Quantification of four different mitochondrial architectures within the cell: tubular, mixed (tubular and vesicular), vesicular, and fragmented. **(C)** Average number of mitochondria per cell in all the clones. Error bars indicate the standard deviation. Student’s *t*-test, FXN-138.1 *p* = 0.00006, FXN-138.2 *p* = 0.00004 versus pLKO.1-NT. **(D)** Mitochondrial length distribution in all the clones. We observed fewer mitochondria but higher mitochondrial length in frataxin-deficient cells than in control. Electron microscopy images of pLKO.1-NT control **(E)** and clone FXN-138.1 **(F,G)** show normal and long tubular mitochondria, respectively (*). Clone FXN-138.1 **(G)** apparently shows increased mitochondrial fusion morphology. Frataxin-deficient cells appeared to have higher lysosomal content (black arrow), with residual bodies (boxed) and vacuoles (white arrow). ***p ≤ 0.001.

Maintenance of the mitochondrial network is fundamental for the normal function of mitochondria and their adaptation to different metabolic and energetic needs of different tissues in homeostasis and disease. Mitochondrial division is usually associated with apoptosis (Frank et al., 2001); in contrast, mitochondrial fusion protects cells from apoptotic cell death (Olichon et al., 2003). As the silenced clones expressed a senescent phenotype with no evidence of apoptosis, we wanted to know the dynamics of the mitochondrial network. As expected, we did not observe a fragmented pattern. On the contrary, frataxin-depleted cells showed a fusion network with tubular morphology (**Figures 7A,B**), a reduced number of mitochondria (**Figure 7C**; Student’s *t*-test; FXN-138.1 *p* = 0.00006, FXN-138.2 *p* = 0.00004), and an increase of the number of large organelles as the major pattern (**Figure 7D**). Electron microscopy images are consistent with the tubular pattern observed. A qualitative assessment showed small round mitochondria in control clone (**Figure 7E**), and tubular pattern and altered morphology with swollen mitochondria are observed in clone FXN-138.1 (**Figures 7F,G**).

### FRATAXIN DEFICIENCY IS ASSOCIATED WITH INCREASED BASAL AUTOPHAGY

**FIGURE 8:**
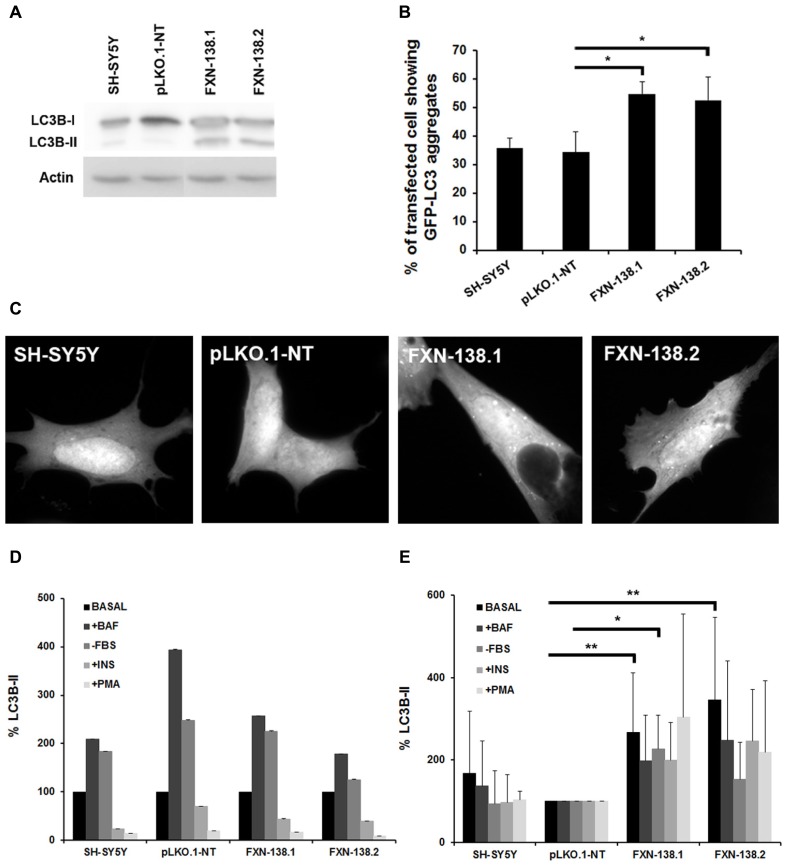
**Effects of frataxin deficiency on the autophagy process. (A)** Western blotting using anti-microtubule chain 3 (LC3) antibody. We detected LC3-I conversion and LC3-II turnover. The figure shows an increase level of LC3-II in *FXN*-deficient cells. Results are representative of five experiments. Proteins were quantified for each lane using Fujifilm’s Multi-Gauge Software. To allow for loading variation, values were normalized to the actin control. **(C)** Cells were transfected with EGFP-LC3 and, after culturing for an additional 24 h in complete medium, were examined by confocal fluorescence microscopy. Frataxin depletion increased GFP-LC3 puncta in SH-SY5Y cells, while puncta were not found in GFP control cells. **(B)** The percentage of cells showing GFP-LC3 aggregates was quantified. Data are means ± standard deviations from three independent experiments. At least 300 cells were analyzed for each. Student’s *t*-test, FXN-138.1 *p* = 0.021, FXN-138.2 *p* = 0.043 versus pLKO.1-NT. **(D)** The autophagyc response was assessed by culturing cells in different conditions: with 0.1 μM bafilomycin A1, in the absence of serum, with 100 nM insulin, or with 1 μM PMA. LC3-II quantification showed no alteration of autophagyc flux in any clone when compared versus itself in the basal condition. **(E)** *FXN*-deficient cells presented elevated levels of the autophagy marker in every condition with respect to control cells (pLKO.1-NT). Baseline condition (Basal), plus bafilomycin (+BAF), without fetal bovine serum (-FBS), plus insulin (+INS), plus phorbol 12-myristate 13-acetate (+PMA). Student’s *t*-test was applied for statistics. Both FXN-138.1 (*p* = 0.003) and FXN-138.2 (*p* = 0.002) shown statistical differences in basal condition versus pLKO.1-NT. After FBS starvation only the FXN-138.1 clone (*p* = 0.013) shown statistical differences. *p ≤ 0.05; **p ≤ 0.01.

Autophagy has a cardinal role in cellular quality control and is involved in oxidative stress-induced adaptation, as a defensive mechanism against oxidative injuries (Chu, 2006). In our frataxin deficiency cell model, we observed several phenomena that might trigger autophagy, such as the decrease of OXPHOS activities and decline of the ΔΨm. Moreover, the accumulation of numerous lysosomal vesicles observed by electron microscopy could be related to the increase of basal autophagy (**Figure 7F**). Based on these findings, we investigated the balance of the autophagy pathway in frataxin-depleted clones. We measured the expression of LC3-II as a major regulator of autophagosome formation in both baseline conditions and after induction or inhibition of the autophagy signaling pathway. In baseline conditions, frataxin-deficient cells showed an increase of both LC3-II expression, as measured by Western blot analysis (**Figure 8A**), and the number of cells containing GFP-LC3 puncta (**Figures 8B,C**), which indicated autophagy induction.

Then, we investigated the cellular status of the autophagic pathway. We induced autophagosome formation by adding bafilomycin A1, which blocks the fusion of autophagosomes with lysosomes and prevents the degradation of LC3-II, or by the removal of serum in cultures. We also inhibited autophagy by either insulin or PMA, which stimulate the mTORC1 pathway that further negatively regulates autophagy. We observed that WT cells, pLKO.1-NT control cells and knockdown clones responded properly to the induction of autophagosome formation and to autophagy inhibition (**Figure 8D**). These findings suggested that the reduction of cellular frataxin levels did not affect the autophagy machinery *per se*. Interestingly, when data from knock-down clones were compared to those from the pLKO.1-NT clone we observed that depletion of frataxin was associated with significant baseline increase of LC3-II, a trend that was also observed for the other experimental conditions (**Figure 8E**). This finding suggest that these cells could be more susceptible to an increase in autophagy activity against cellular insults such as oxidative stress or bioenergetic deficit, and that autophagy might be cytoprotective in cells when frataxin is depleted.

### FRATAXIN DEPLETION CAUSES ENDOPLASMIC RETICULUM STRESS

**FIGURE 9:**
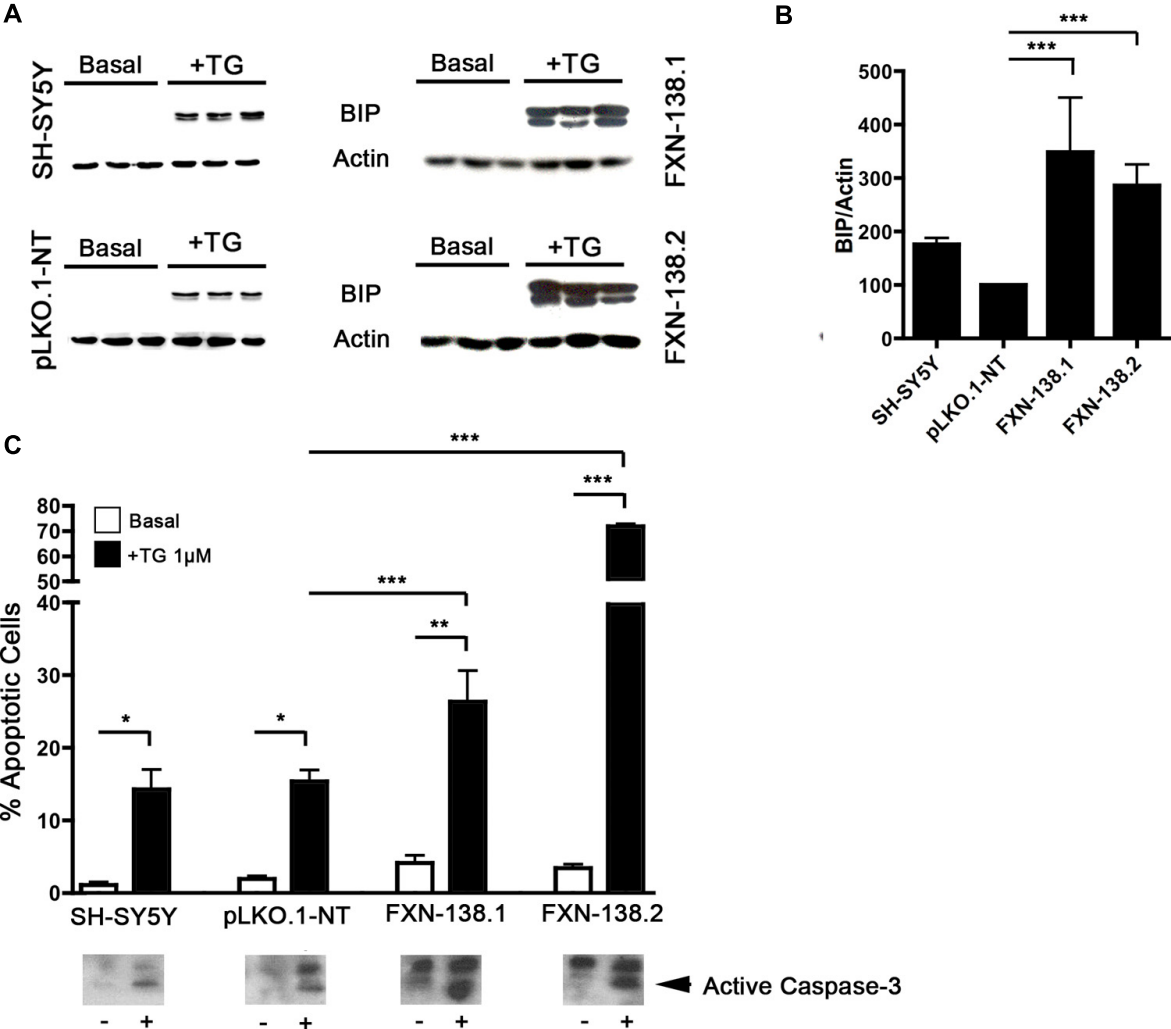
***FXN* depletion results in increased endoplasmic reticulum (ER) stress. (A)** Western blot analysis of ER stress marker BIP in control and FXN-deficient cells in three independent samples. **(B)** Normalized intensities expressed as a percentage of the BIP intensity shown in **(A)**. Actin was used as a loading control. The columns and bar show the mean and standard deviation. Student’s *t*-test, FXN-138.1 *p* = 0.008, FXN-138.2 *p* = 0.023 versus pLKO.1-NT. **(C)** Quantification of apoptotic cells by flow cytometry. Untreated cells (white bars) and cells treated with thapsigargin (TG, black) were fixed and stained with propidium iodine. The subG1 population was quantified at least in three independent experiments. Bars show mean ± standard deviation. Student’s *t*-test was applied for statistics. Every cell types were compared between basal condition and with TG: SH-SY5Y *p* = 0.16, pLKO.1-NT *p* = 0.042, FXN-138.1 *p* = 0.032, FXN-138.2 *p* = 0.001. Comparison after TG treatment between pLKO.1-NT and FXN-138.1 *p* = 0.050, and FXN-138.2 *p* = 0.00004. The activation of caspase-3 was tested by western blot (lower panel) using specific antibody in resting conditions (-) and TG-treated cells (+). *p ≤ 0.05; **p ≤ 0.01; ***p ≤ 0.001.

The stress response is a major signaling pathway that regulates autophagy. The depletion of frataxin in SH-SY5Y cells was associated with increased susceptibility to autophagy, but also with the presence of oxidative stress. In order to deepen into the characterization of the cellular stress status, we investigated the ER stress pathway, as it is stimulated by ROS but also upregulates autophagy. To determine the effect of frataxin silencing on ER stress, we analyzed levels of the unfolded protein response (UPR)-related chaperone BiP/Grp78. This protein was not increased in baseline conditions; however, a significant increase of BiP/Grp78 expression was obtained in frataxin-depleted cells after treatment with thapsigargin (**Figures 9A,B**).

Under conditions of prolonged stress signals, the UPR can induce apoptosis (Rao et al., 2004). In our models, we observed that the level of cell death induced by ER stress was related to frataxin levels. Thapsigargin induced cell death in both control and knockdown clones, but it was significantly higher in the silenced clones suggesting that reduction of frataxin levels increases ER stress susceptibility (**Figure 9C**). To confirm whether the increase in cell death is related to UPR-apoptotic activation, we analyzed caspase-3 activation by Western blot (**Figure 9C**). We found strong caspase-3 activation in thapsigargin treated cells. Thus, a prolonged exposure to ER stress of frataxin-depleted cell induces apoptotic cell death.

### FRATAXIN DEFICIENCY AFFECTS MITOCHONDRIAL Ca^2+^ UPTAKE CAPACITY

**FIGURE 10:**
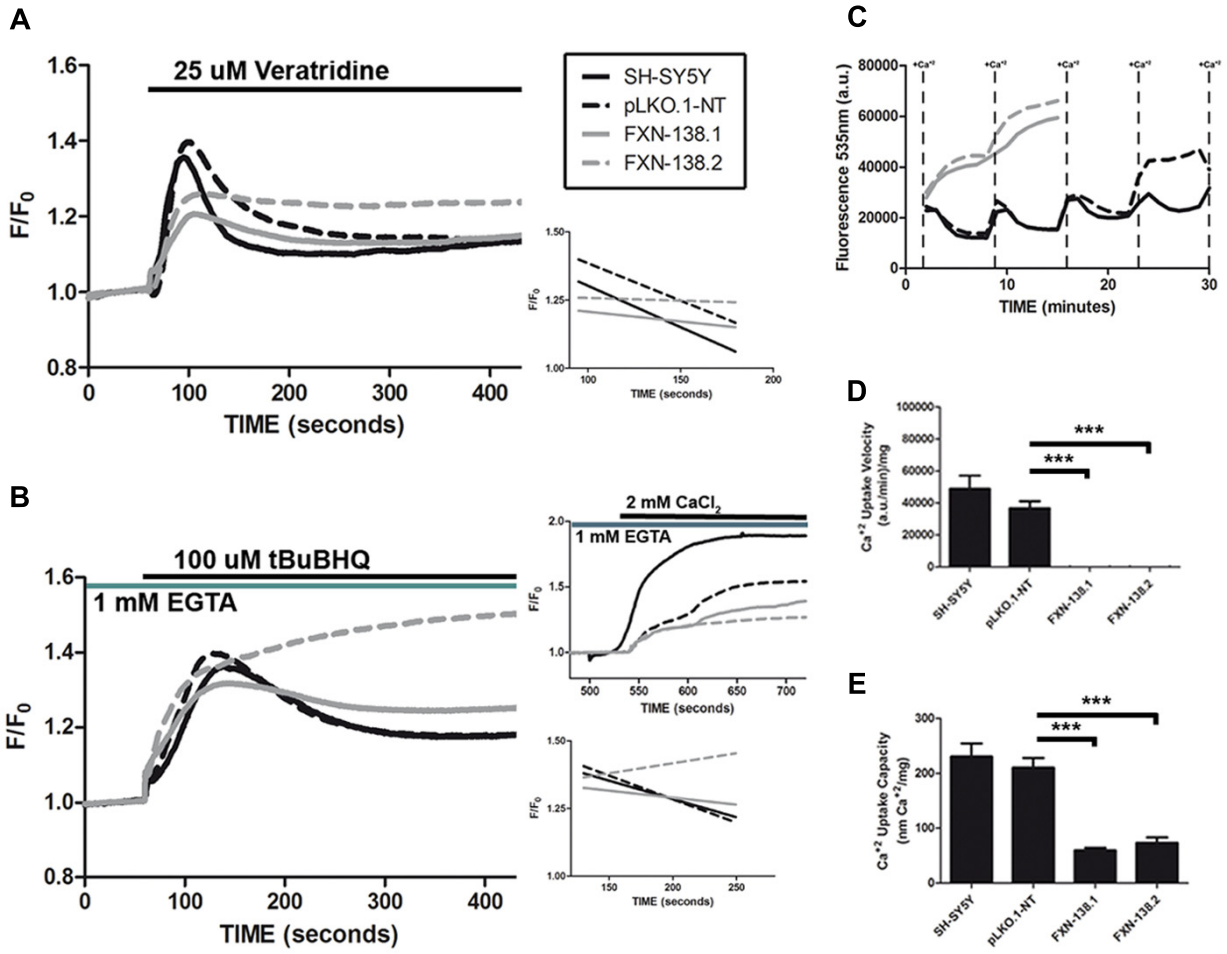
**Calcium homeostasis in frataxin-depleted SH-SY5Y cells. (A)** Changes in fura-2 [Ca^2+^]_cyt_ fluorescence intensity after addition of 25 μM veratridine. The recovery slopes for each clone after calcium entry (right, lower panel) show the difference in behavior for calcium buffering between control and frataxin-depleted clones. Traces represent means of no fewer than 100 cells from at least five experiments. **(B)** Changes in fura-2 [Ca^2+^]_cyt_ fluorescence intensity after addition of 100 μM tBuBHQ in Ca^2+^-free medium. The recovery slopes for each clone after emptying of calcium from the endoplasmic reticulum (right, lower panel) show the different behavior for calcium buffering between control and frataxin-depleted clones. The activation of SOCE with the addition of 2 mM Ca^2+^ to cells treated with 100 μM tBuBHQ in Ca^2+^-free medium is altered in frataxin-depleted cells (right, upper panel) as the activation of the process is lower than in the control cells. Traces represent means of no fewer than 100 cells from at least three experiments. **(C)** Changes in Calcium Green-5N fluorescence in digitonin-permeabilized cells after the addition of pulses of 4 nmol of Ca^2+^. Traces represent means of at least four experiments for each clone. The Ca^2+^ uptake velocity [**(D)**; Student’s *t*-test, FXN-138.1 *p* = 0.0004, FXN-138.2 *p* = 0.0004 versus pLKO.1-NT] and capacity [**(E)** ;Student’s *t*-test, FXN-138.1 *p* = 0.0002, FXN-138.2 *p* = 0.0002 versus pLKO.1-NT] rates were calculated from the uptake slopes in **(C)**. ***p ≤ 0.001.

Inter-organelle communication between the mitochondria and ER affects mitochondrial distribution and dynamics and is relevant for the proper physiology of several metabolic and signaling pathways. Interaction occurs at the mitochondria-associated membranes (MAMs) that create specific environments for the localization and function of molecules that participate in cell metabolism such as calcium homeostasis (Marchi et al., 2013). ER is the major cellular structure for Ca^2+^ storage and proper ER-mitochondria communication supports Ca^2+^ release and buffering (Cali et al., 2012). Since frataxin deficiency induced significant changes of mitochondrial physiology and ER stress, calcium metabolism and mitochondrial buffering might be disturbed in the knockdown clones. To test this possibility, we used agents that increase cytosolic Ca^2+^and mobilize ER-Ca^2+^. Veratridine increases intracellular Ca^2+^ by opening voltage-dependent Na^+^ channels and then voltage-activated calcium channels. tBuBHQ is an inhibitor of SERCA, the ER Ca^2+^-ATPase that allows net Ca^2+^ leak from the ER. The emptying of ER-Ca^2+^ stores activates Ca^2+^ entry through low-conductance plasmalemmal channels that are regulated by SOCE (Putney and Thomas, 2006). The Ca^2+^ buffer capacity, measured as the differences between the maximum calcium peak and the stabilization of the response showed a defect in frataxin depleted clones (**Figure 10A**). Moreover, the percentage of Ca^2+^ levels recovery, measured as the different between maximum and minimum fluorescence during veratridine induction, suggested again a problem in buffering capacity. Whereas both WT and pLKO.1-NT cells restored [Ca^2+^]_cyt_ levels after veratridine treatment (70 and 67% respectively), frataxin-deficient cells failed to recover normal Ca^2+^ cytosolic concentrations (37% for FXN-138.1 and 11% for FXN-138.2). Thus, we investigated Ca^2+^ release from the ER by measuring [Ca^2+^]_cyt_ in Ca^2+^-free medium and inhibition of the SERCA pump by tBuBHQ. Again, FXN-silenced clones did not show reduced calcium levels after Ca^2+^ release from the ER (**Figure 10B**). The percentages of Ca^2+^ levels recovery were 49% for SH-SY5Y and 53% for pLKO.1-NT versus 22% for FXN-138.1 and absent in FXN-138.2, confirmed abnormal buffering of intracellular Ca^2+^ levels. Mitochondrial buffering is crucial to properly calcium influx by store-operated calcium channels. The induction of SOCE by ER-calcium released was reduced in the frataxin silenced cells compared to control clones (**Figure 10B**, upper right panel). To test whether the abnormal Ca^2+^ buffering in *FXN*-silenced clones was due to mitochondrial buffering capacity dysfunction, we performed Ca^2+^ uptake capacity experiments in digitonin-permeabilized cells in the presence of Calcium Green 5N. In contrast to WT and control cells, *FXN*-silenced cells were unable to take up Ca^2+^ in the mitochondria, which indicated a strong defect in Ca^2+^ buffering by mitochondria deficient for frataxin (**Figures 10C–E**). Then, an excess of local [Ca^2+^]_cyt_ in frataxin deficient cells due to the inability of mitochondria to uptake calcium, might induce a negative feedback of the SOCE mechanism.

## DISCUSSION

In order to investigate the effects of a lack of frataxin on cellular homeostasis, we developed a cell model of frataxin deficiency by *FXN* gene silencing in the human neuroblastoma cell line SH-SY5Y. The main advantage of this model is that neuroblastoma cells are derived from the neural crest, which is the developing structure of the nervous system that gives rise to neurons from the DRG, the major target structure involved in FRDA neuropathology. The two knockdown cell clones, FXN-138.1 and FXN-138.2, showed maintained reduction of frataxin over time, simulating a chronic deficiency.

Frataxin silencing induced slow cell growth, similar to those reported in several cellular models of frataxin deficiency (Zanella et al., 2008; Calmels et al., 2009; Lu et al., 2009). In our cell model, slow growth was not associated with apoptotic cell death but with cellular senescence, as demonstrated by increased SA-βgal activity and cell cycle arrest at the G1 phase. We have also found that frataxin deficiency is associated with mitochondrial elongation and, interestingly, morphodynamic changes of mitochondria closely link to the cell cycle (Lee et al., 2013) and mitochondrial elongation concretely triggers cellular senescence (Lee et al., 2007).

The relationship between frataxin deficiency and apoptosis remains unclear. Cortopassi and colleagues (Lu et al., 2009) in their study of the effect of frataxin silencing in several neural cell lines observed that only one Schwann cell-derived line exhibited viability decrease. By contrast, cell death associated with frataxin deficiency has been reported in a neuroblastoma cell line (Palomo et al., 2011) and rat DRG neurons (Mincheva-Tasheva et al., 2013). In the SH-SY5Y cells, large-scale apoptosis was observed after neuron-like differentiation, but not if differentiation was not induced (Palomo et al., 2011). We think that the different apoptotic response may be due to differential effects of permanent frataxin silencing versus transitory depletion. Maintained depletion may favor cell adaptation, which could promote molecular mechanisms to prevent cell death, such as autophagy.

To determine the biological status of these senescent cells and the pathogenic mechanisms associated with frataxin deficiency, we investigated several mitochondrial and cellular functions and pathways. We observed that the partial lack of frataxin provoked changes in mitochondrial function and network dynamics, induced the increase of the autophagy threshold, induced cellular stress, and affected normal calcium metabolism management by the mitochondria.

Energy depletion and inner membrane potential depolarization, with a reduction of complex IV activity, suggest malfunction of the ETC and oxidative phosphorylation. Alterations of the respiratory chain activities associated with frataxin deficiency have been described but the biological consequences depend on the cell models and patient tissues investigated. Reduced activities of ETC complexes have been observed (Rotig et al., 1997; Bradley et al., 2000; Gonzalez-Cabo et al., 2010), but there is no evidence of ETC involvement in patients’ nervous system (Bradley et al., 2000), conditional knockout mice (Puccio et al., 2001), or neurons derived from reprogrammed iPS cells (Hick et al., 2013). Mitochondria are the major source of ROS production, mainly the superoxide anion $O_{2}^{•-}$ and H_2_O_2_. The increase of $O_{2}^{•-}$ radical and protein carbonylation we observed in *FXN*-silenced clones agrees with previous reports (Gonzalez-Cabo and Palau, 2013) and confirms the role of oxidative stress in the pathogenesis of frataxin deficiency.

The maintenance and proper connection of both function and morphology are fundamental in mitochondrial homeostasis (Nunnari and Suomalainen, 2012). In fact, an imbalance between fusion and fission activities results in dysfunctional mitochondria. Frataxin knockdown cells showed a tubular pattern that indicates fusion activity of the mitochondrial network. However, in the same way, the induction of fusion should be associated with high OXPHOS activity and an increase in mitochondrial membrane potential, which were not observed in frataxin-depleted cells. This apparent contradictory response of mitochondrial physiology may be related to an attempt of the mitochondrial network to buffer respiratory defects and oxidative stress (Chen et al., 2005, 2007). Hyperfused mitochondria should protect cells from apoptotic cell death, which agrees with our observation in the knockdown clones. The increased expression of the autophagosome marker LC3-II in knockdown cells suggests that the reduction of frataxin induces autophagy in basal conditions. Autophagy may be induced by a wide variety of cellular stresses, including nutrient deprivation, high bioenergetic demands, changes in redox metabolism with the production of ROS, infection, protein aggregate accumulation, and hypoxia (Scherz-Shouval and Elazar, 2007; Kroemer et al., 2010; Murrow and Debnath, 2013). Pharmacological analysis of the autophagy pathway by inhibition with bafilomycin A1 or nutrient starvation, or by induction of the mTORC1 pathway with either insulin or PMA confirmed that autophagy signaling functioned properly in frataxin deficiency. Thus, the observed over active autophagy in frataxin-depleted cells might be the consequence of a cytoprotective response to abnormal mitochondrial homeostasis manifested by the reduction of cell respiration and ΔΨm and the induction of oxidative stress.

The whole metabolic scenario triggered by the partial reduction of frataxin in SH-SY5Y cells relates mitochondrial dysfunction and fusion, cellular response to stress, and mitochondrial Ca^2+^ homeostasis. The incapacity of the mitochondria to buffer Ca^2+^ release might be due to inner membrane depolarization and the changes in redox metabolism associated with abnormal ROS production. Both oxidative stress and low cytosolic Ca^2+^ buffering by the mitochondria may induce other cellular stress responses such as ER stress, but not apoptosis unlike other authors have related (Mincheva-Tasheva et al., 2013). These pathological responses may finally trigger the autophagy response in an attempt to avoid cytotoxicity and cell death. Evidence of autophagy involvement in frataxin deficiency has been observed in some FRDA mouse models. Histological study of the DRG showed degeneration of large sensory neurons, with the presence of vacuoles and lipofucsin accumulation, both lesions related to the autophagy process (Simon et al., 2004; Al-Mahdawi et al., 2006). We think that autophagy has a protective function for maintaining cellular health in frataxin-deficient cells, since the cells are able to eliminate the mitochondrial damage and oxidized proteins to survive, even though the autophagy response is not enough to avoid ER stress-induced apoptosis. Silencing of the *FXN* gene induced ER stress after depletion of ER Ca^2+^ stores by the inhibition of SERCA pumps. Moreover, ER stress promoted more apoptosis in knockdown clones than control cells. In addition, frataxin-depleted cells failed to buffer Ca^2+^ properly, as a consequence of reduced Ca^2+^ uptake capacity of the mitochondria that could explain the negative feedback of SOCE mechanism by the excess of local cytosolic calcium. Thus, it is plausible that the apoptosis induced by ER stress may be the consequence of anomalous Ca^2+^ management by the mitochondria. Such Ca^2+^-induced cell toxicity could be eliminated by normalizing intracellular calcium (Wong et al., 1999).

Cellular senescence is a program executed by cells in response to a variety of stresses, mainly associated with cancer and aging (Rodier and Campisi, 2011). Based on the biological phenotype of our cellular model of frataxin depletion, we think that the cells had acquired a senescent phenotype to avoid death cell. Cellular senescence has not been previously described in FRDA patients or disease models. How could cellular senescence contribute to the pathophysiology of disease? Cell senescence entails a loss of functionality, and hence the appearance of an age-related phenotype or early aging. Recently, cellular senescence has been assigned a direct role in embryonic development (Munoz-Espin et al., 2013). Thus, the loss of cellular functionality could affect the neurogenesis program and alter proper development of the peripheral nervous system. In their recent necropsy studies, Koeppen and Mazurkiewicz (Koeppen and Mazurkiewicz, 2013) have suggested that FRDA neuropathology may be interpreted as a combination of developmental delay and degeneration. These authors observed that major lesions in the central nervous system involving the dentate nucleus, Betz cells, and corticospinal tracts showed atrophy and degeneration. This is, however, in contrast to DRG, which are the subject of hypoplasia and superimposed atrophy (Koeppen and Mazurkiewicz, 2013). We propose that whereas apoptotic cell death may be associated with neurodegeneration, the senescent phenotype we observed could be a phenomenon associated with hypoplasia and developmental delay, supporting the hypothesis proposed by Koeppen and Mazurkiewicz (2013) that DRG are subject to hypoplasia and superimposed atrophy.

The study in depth of and mitochondrial functions and pathways such as bioenergetics and cellular redox status, has established that frataxin deficiency causes mitochondrial failure. In this paper we introduce new pathways and cellular processes related to mitochondria biology, calcium homeostasis and ER that have been related to neurodegeneration (Schon and Przedborski, 2011) but so far have not with FRDA pathogenesis. Our data suggest that the lack of frataxin may induce cellular senescence, changes in Ca^2+^ homeostasis and ER stress, which participate in the generation of FRDA pathogenesis and neuropathology. These mechanisms deserve additional exploration as potential therapeutic targets for the treatment of FRDA patients.

## Conflict of Interest Statement

The authors declare that the research was conducted in the absence of any commercial or financial relationships that could be construed as a potential conflict of interest.
